# THE MEDIATING ROLES OF HEALTH LOCUS OF CONTROL AND COPING ON THE PAIN-DEPRESSION PATHWAY

**DOI:** 10.1093/geroni/igz038.579

**Published:** 2019-11-08

**Authors:** Kyrsten Costlow, Patricia A Parmelee, Tina Harralson

**Affiliations:** 1 University of Alabama, Tuscaloosa, Alabama, United States; 2 The University of Alabama, Tuscaloosa, Alabama, United States; 3 Tridiuum, Philadelphia, Pennsylvania, United States

## Abstract

The literature on health locus of control (HLC) suggests that individuals who believe that their health is internally determined are more likely to use active coping strategies than those who believe their health is determined by chance or powerful others (Brosschot, Gebhardt, & Godaert, 1994; Gibson & Helme, 2000). Coping strategies (Klapow et al., 1995) and HLC (Campbell, Hope, & Dunn, 2017) have been found to influence the relation between chronic pain and depression. We hypothesized that the relation between osteoarthritis pain and depression would be serially mediated by HLC and coping. Self-report measures of osteoarthritis pain (Meenan, Mason, Anderson, Guccione, & Kazis, 1992; Parmelee, Katz, & Lawton), HLC (Wallston, Wallston, & DeVellis, 1978), coping strategies (Felton & Revenson, 1984; Rosenstiel & Keefe, 1983), and depression (Radloff, 1977) were examined in 367 older adults with osteoarthritis of the knee. Hayes’ (2013) PROCESS macro was used to test the hypothesized serial multiple mediation for three subscales of HLC: internality (IHLC), chance (CHLC), and powerful others (PHLC). After controlling for age, the hypothesized serial mediation was statistically significant for IHLC and CHLC but not PHLC. More specifically, osteoarthritis pain significantly increased CHLC, which increased negative coping and depression in turn. Osteoarthritis pain significantly decreased IHLC, which was associated with both positive and negative coping strategies in a complex serial mediation. These findings suggest that interventions targeting HLC and/or coping strategies may be able to alter the pain-depression pathway for older adults with chronic osteoarthritis pain. (Supported by R01-MH51800, P. Parmelee, PI).

